# Supplementary material for: Overexpression of human BAG3P209L in mice causes restrictive cardiomyopathy
Source: Nat Commun. 2021 Jun 11;12:3575. doi: 10.1038/s41467-021-23858-7 (PMC8196106; doi:10.1038/s41467-021-23858-7)
Supplement: Supplementary file 1 — Supplementary Information [file 41467_2021_23858_MOESM1_ESM.pdf]

Overexpression of human BAG3^{P209L} in mice causes restrictive cardiomyopathy

Kenichi Kimura, Astrid Ooms, Kathrin Graf-Riesen, Maithreyan Kuppusamy, Andreas Unger, Julia Schuld, Jan Daerr, Achim Lother, Caroline Geisen, Lutz Hein, Satoru Takahashi, Guang Li, Wilhelm Röll, Wilhelm Bloch, Peter F.M. van der Ven, Wolfgang A. Linke, Sean M. Wu, Pitter F. Huesgen, Jörg Höhfeld, Dieter O. Fürst, Bernd K. Fleischmann, Michael Hesse

Supplementary Information

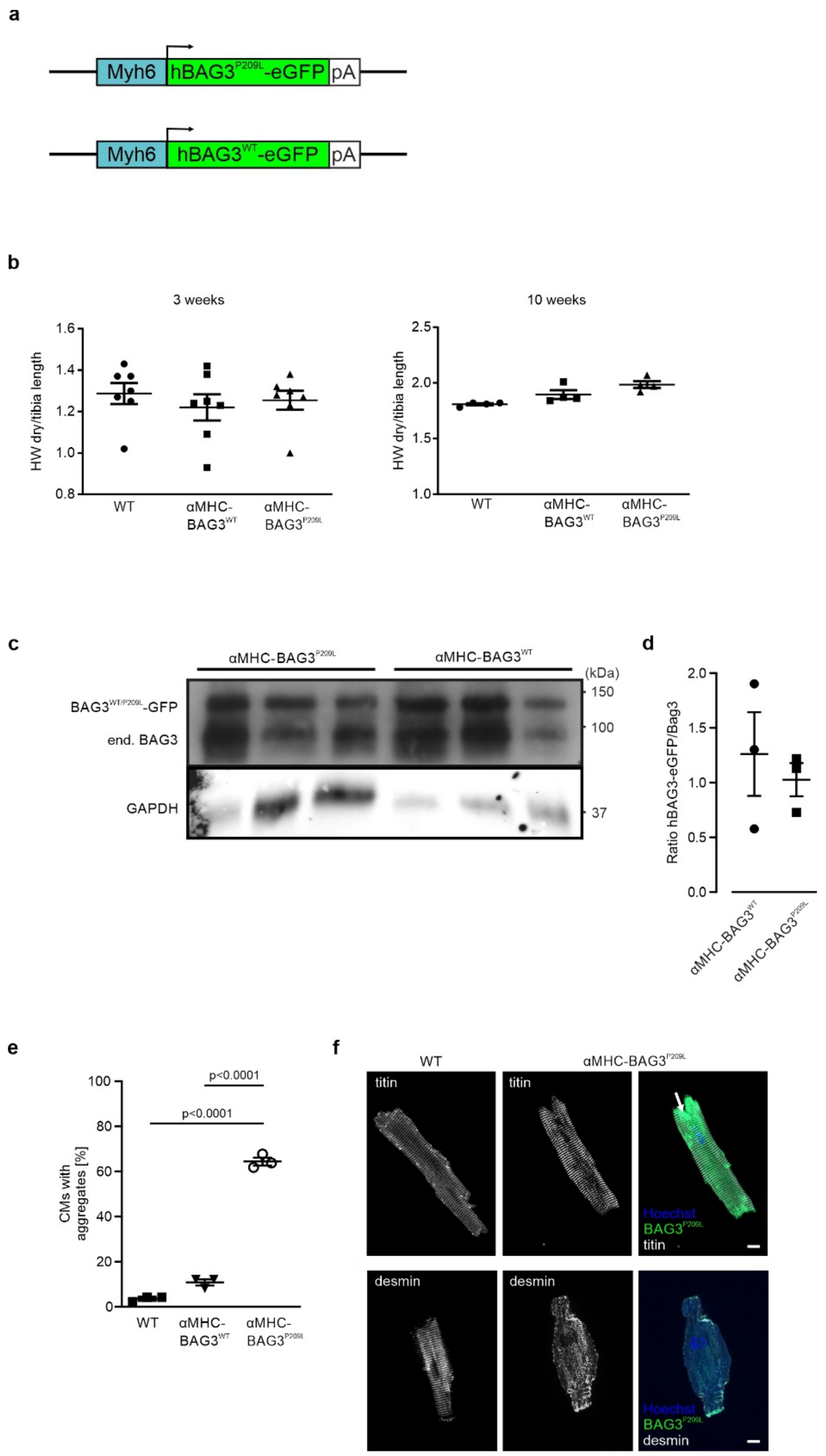

Supplementary Figure 1: Characterization of αMHC-BAG3^{WT}-eGFP, and αMHC-BAG3^{P209L}-eGFP transgenic mice

a Expression construct used for the generation of αMHC-BAG3^{WT}-eGFP, and αMHC-BAG3^{P209L}-eGFP transgenic mice. **b** Heart weight (HW) to tibia length is unchanged in 3- and 10-week-old

WT, αMHC-BAG3^{WT}-eGFP, and αMHC-BAG3^{P209L}-eGFP mice. Mean ± SEM, n = 4 mice per group, One-way ANOVA. **c**,**d** Immunoblots from Langendorff-isolated eGFP^{+} CMs from αMHC-BAG3^{WT} and αMHC-BAG3^{P209L} mice **c** and quantitation of the ratio of hBAG3^{P209L} to mouse Bag3 based on immunoblot data **d**. EGFP^{+}-CMs from αMHC-BAG3^{WT} and αMHC-BAG3^{P209L}-mice have nearly as much human BAG3, as mouse Bag3; expression normalized to GAPDH levels. Mean ± SEM. n = 3 hearts per group. Two-sided Student´s T-test. **c**. **e**, **f** Percentage of Langendorff-isolated CMs with aggregates from 10-week old mice **e**. Mean ± SEM. n=3 hearts per group. One-way ANOVA. **f** The Z-disc markers titin and desmin display a cross-striated pattern in WT CMs (white). In BAG3^{P209L} expressing CMs titin display a cross-striation pattern, that is lost in vicinity to large BAG3^{P209L} aggregates, whereas desmin partly lost its cross-striated localization and formed small aggregates. BAG3^{P209L} (green) forms large aggregates **f**. Scale bars: 10 µm.

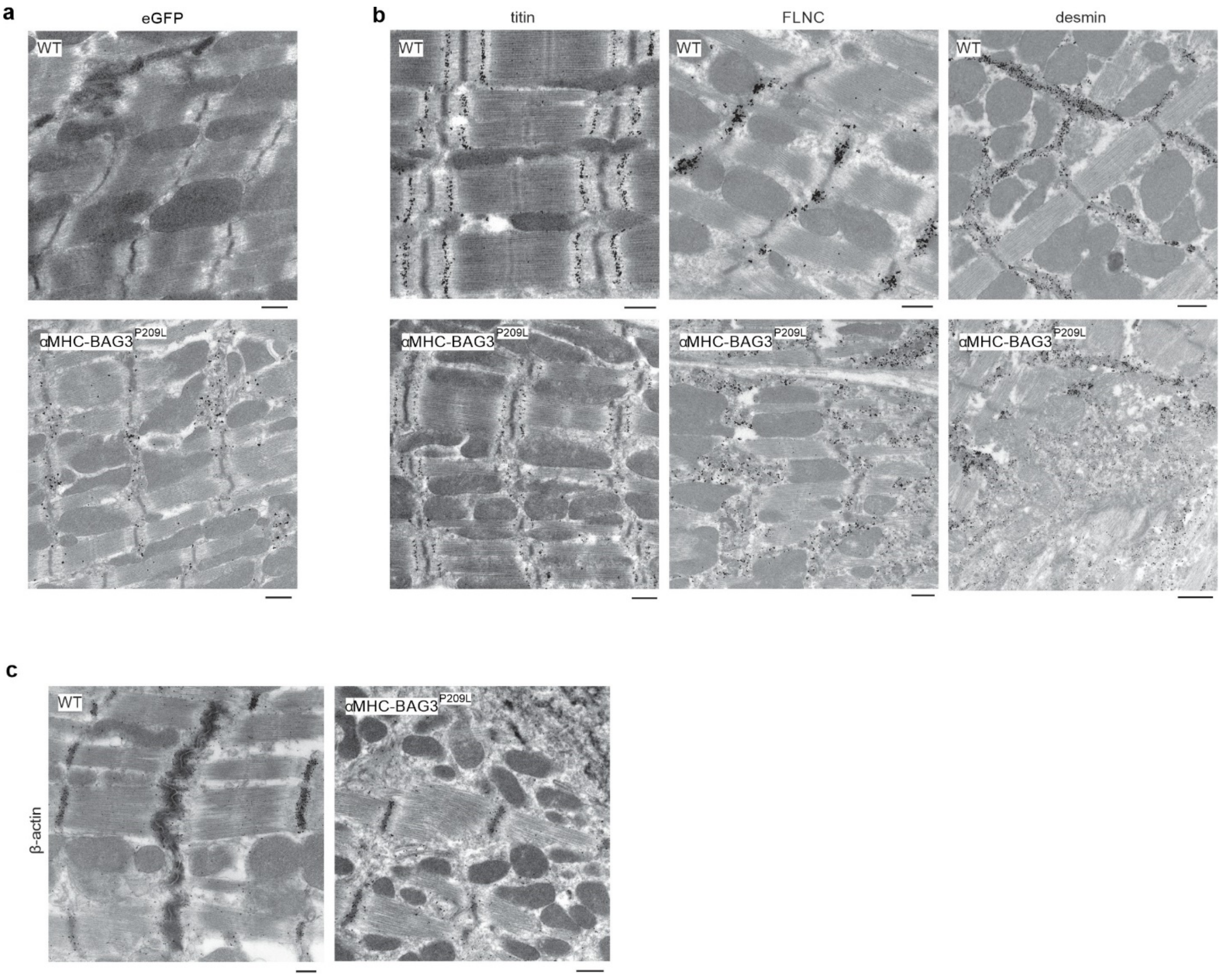

Supplementary Figure 2: Ultrastructural analysis of hBAG3^{P209L}-expressing CMs and immunogold staining for eGFP, titin, FLNC, desmin, and β-actin

a-**c** Immunogold stainings (black dots) of ultrathin sections from WT and αMHC-BAG3^{P209L} mice for eGFP **a** titin **b**, left, FLNC **b**, middle, desmin **b**, right, and β-actin, **c**, analyzed by electron microscopy. BAG3^{P209L}-eGFP, FLNC, and desmin are found in electron-dense aggregates, whereas β-actin is localized exclusively in sarcomeres. The experiments were repeated three times from three independent biological replicates with similar results. WT = wild-type, Scale bars: 1000 nm.

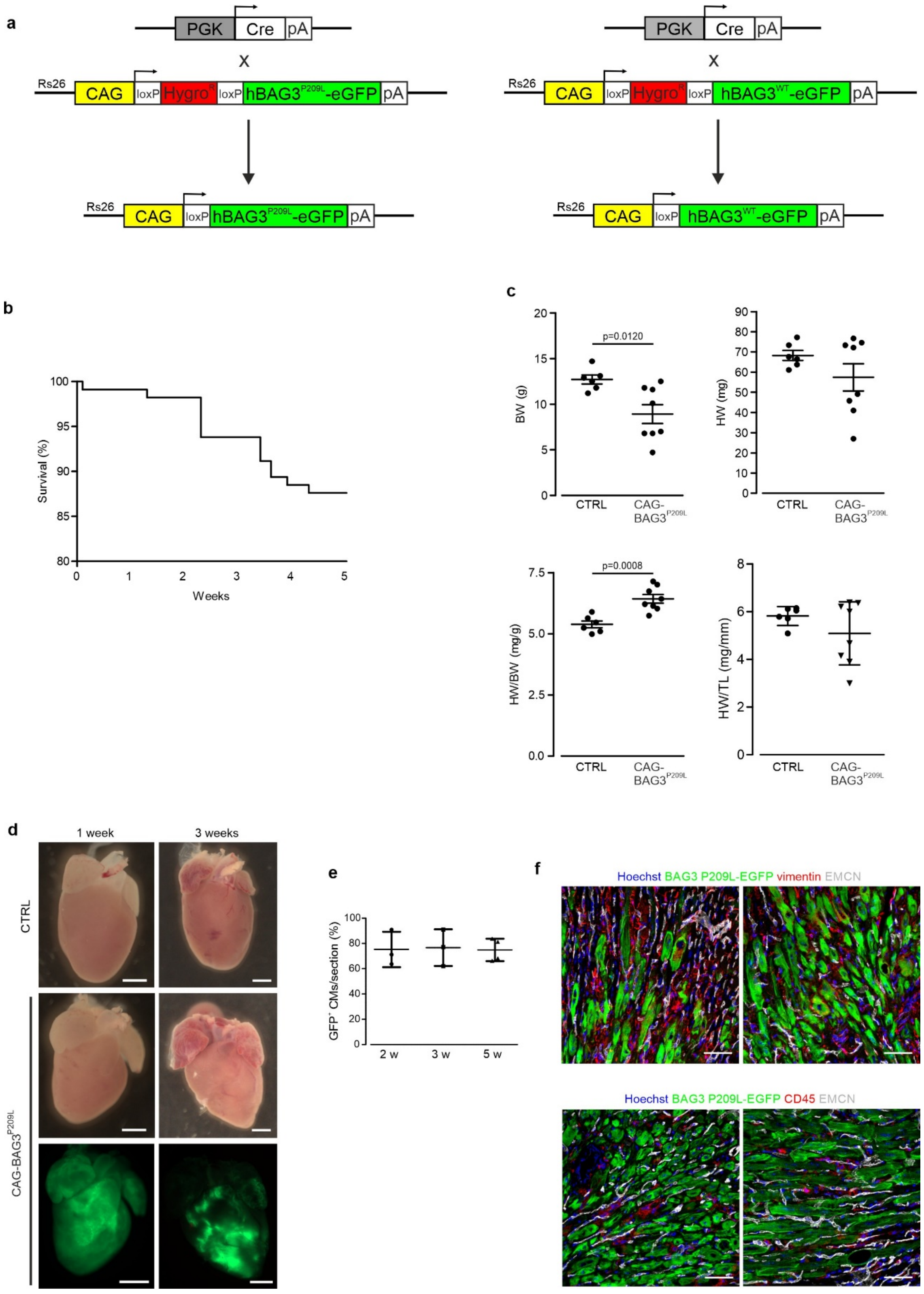

Supplementary Figure 3: Analysis of CAG-BAG3^{P209L}-mice

a Expression constructs used for the generation of CAG-BAG3^{P209L}-eGFP (left), and CAG-BAG3^{WT}-eGFP (right) transgenic mice; breeding schemes for conditional expression of

BAG3^{P209L}-eGFP or BAG3^{WT}-eGFP by crossing with PGK-Cre transgenic mice. **b** Survival curve of CAG-BAG3^{P209L} mice up to 5 weeks of age; n = 113 mice. **c** Body weight (BW) is reduced in CAG-BAG3^{P209L} mice compared to controls (left upper panel), while heart weight (HW, right upper panel), HW/BW (left lower panel), and HW to tibia length (right lower panel) is unchanged at 5 weeks of age. Mean ± SD. n = 6 CTRL and 8 CAG-BAG3^{P209L} mice. Two-sided Student´s T-test. **d** Representative hearts from 2- and 3-week-old CAG-BAG3^{P209L} and CTRL mice; note that CAG-BAG3^{P209L}-hearts display robust BAG3^{P209L}-eGFP expression. The experiments were repeated three times from three independent biological replicates with similar results. Scale bars: 1 mm. **e** Quantification of the percentage of BAG3^{P209L}-eGFP expressing CMs in cardiac sections from 2-, 3-, and 5-week-old CAG-BAG3^{P209L}-mice. Mean ± SEM. n = 3 hearts per group of 2- and 3-week old mice and n = 4 hearts of 5-weeks old mice. **f** Sections of hearts from 5-week-old CAG-BAG3^{P209L}-mice stained for vimentin (red), CD45 (red), and endomucin (EMCN, white). BAG3^{P209L}-eGFP (green) is exclusively expressed in CMs. Scale bars: 50 µm. The experiments were repeated three times from three independent biological replicates with similar results. CTRL = control mice (siblings of CAG-BAG3^{P209L} mice, which are either WT, PGK-Cre, or CAG-flox-hBAG3^{P209L}).

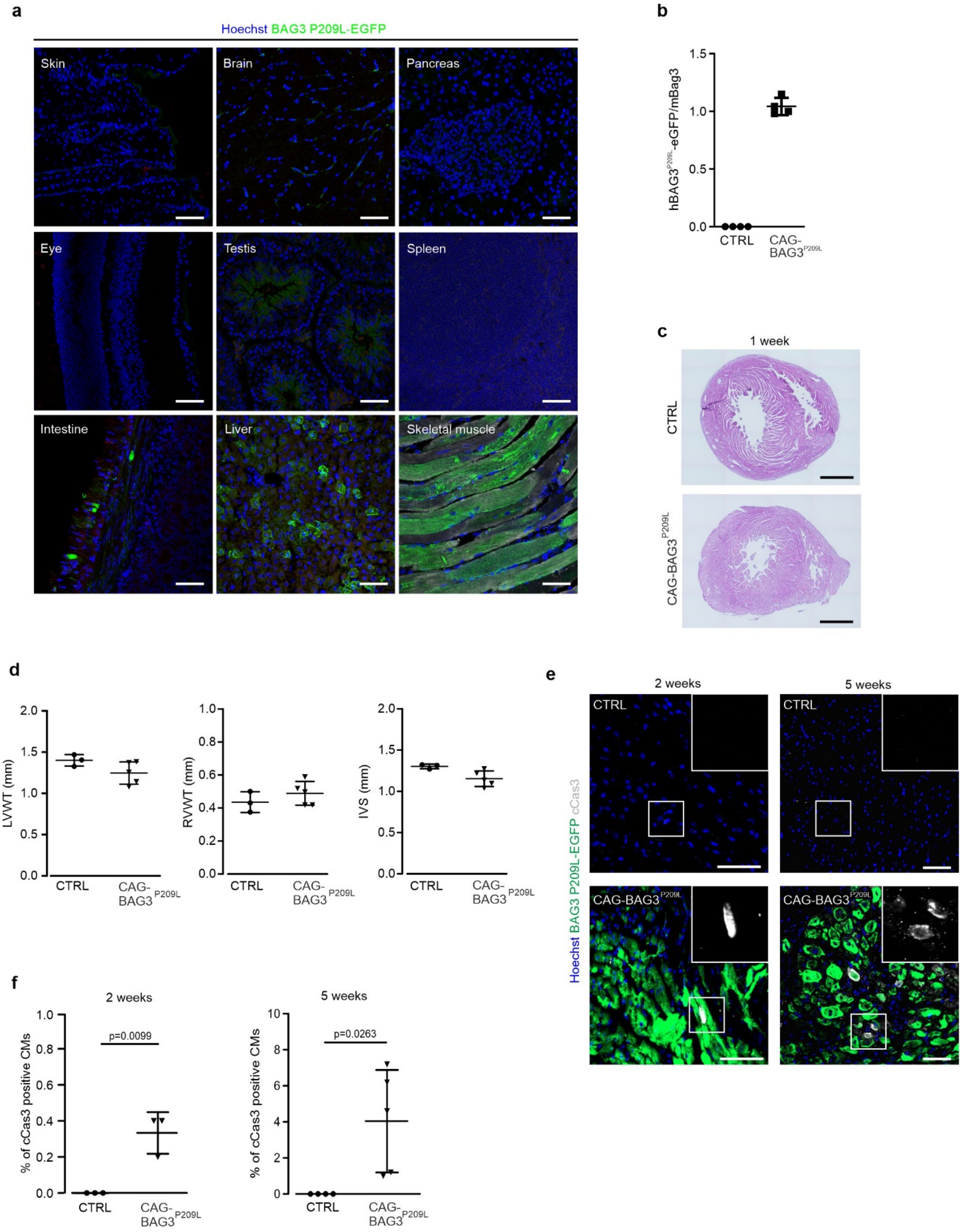

Supplementary Figure 4: Expression pattern of BAG3^{P209L}-eGFP, heart size, and quantification of apoptosis in CAG-BAG3^{P209L} and CTRL mice

a Analysis of the expression of BAG3^{P209L}-eGFP in different tissues revealed that it is almost restricted to striated muscle cells. Scale bars: 50 µm. The experiments were repeated three times from three independent biological replicates with similar results. **b** Ratio of hBAG3^{P209L} to mBAG3 in hearts from CAG-BAG3^{P209L} and CTRL mice. Mean ± SEM. Two-sided Student´s T-test, n = 4 mice per group. **c** HE-stained histological sections of hearts from 1-week old homozygous BAG3^{P209L}-eGFP and CTRL-mice. Scale bars: 1 mm. The experiments were repeated three times from three independent biological replicates with similar results. **d** LVWT (left ventricular wall thickness), RVWT (right ventricular wall thickness), and IVS (*interventricular septal* thickness) of hearts from 5-week-old CAG-BAG3^{P209L} and CTRL-mice, as measured from histological sections. Mean ± SEM. n = 3 CTRL and 5 CAG-BAG3^{P209L} hearts. Two-sided Student´s T-test, CTRL = control. **e** Immunostainings against cleaved Caspase 3 (Cas3) in hearts from 2- and 5-week-old CAG-BAG3^{P209L} and CTRL-mice Scale bars: 50 µm. **f** Percentage of cCas3 positive apoptotic cells in the hearts from 2- and 5-week-old CAG-BAG3^{P209L} and CTRL-mice. Mean ± SEM. n = 3 hearts per group from 2 weeks old mice and n = 4 CTRL and 5 CAG-BAG3^{P209L} hearts from 5 week old mice. Two-sided Student´s T-test. CTRL = control mice (siblings of CAG-BAG3^{P209L} mice, which are either WT, PGK-Cre, or CAG-flox-hBAG3^{P209L}).

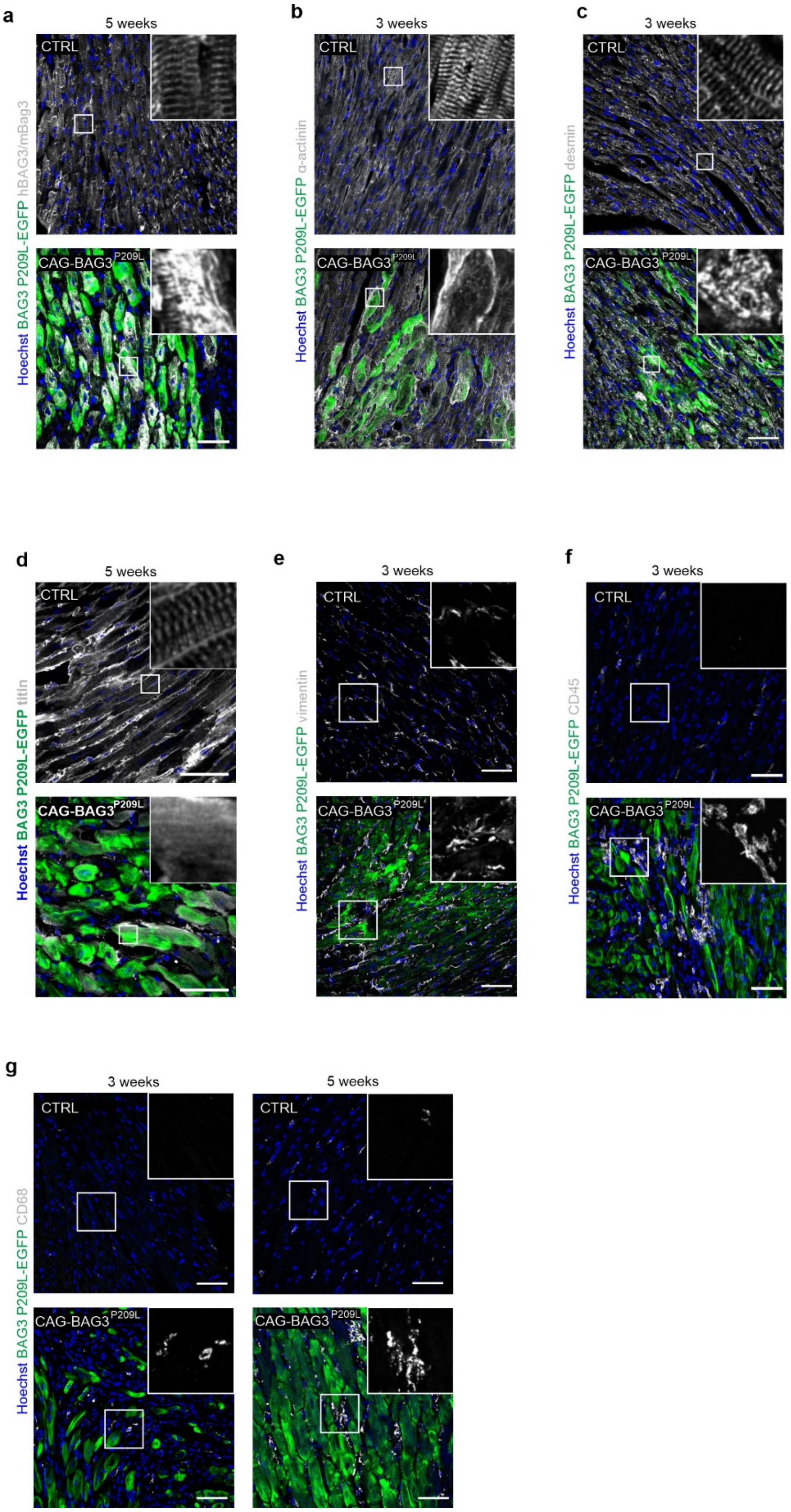

Supplementary Figure 5: Immunofluorescence analysis of CAG-BAG3^{P209L} mouse hearts
a-g Sections of hearts from 3- and 5-week-old CAG-BAG3^{P209L} and CTRL-mice were stained for hBAG3/mBAG3 **a**, α-actinin **b**, desmin **c**, titin **d**, vimentin **e**, CD45 **f**, and CD68 **g**. BAG3^{P209L}-

eGFP (green) forms large aggregates in $hBAG3^{P209L}$, but not in control CMs. Scale bars: 50 μm. The experiments were repeated three times from three independent biological replicates with similar results. CTRL = control mice (siblings of CAG-$BAG3^{P209L}$ mice, which are either WT, PGK-Cre, or CAG-flox-$hBAG3^{P209L}$).

a

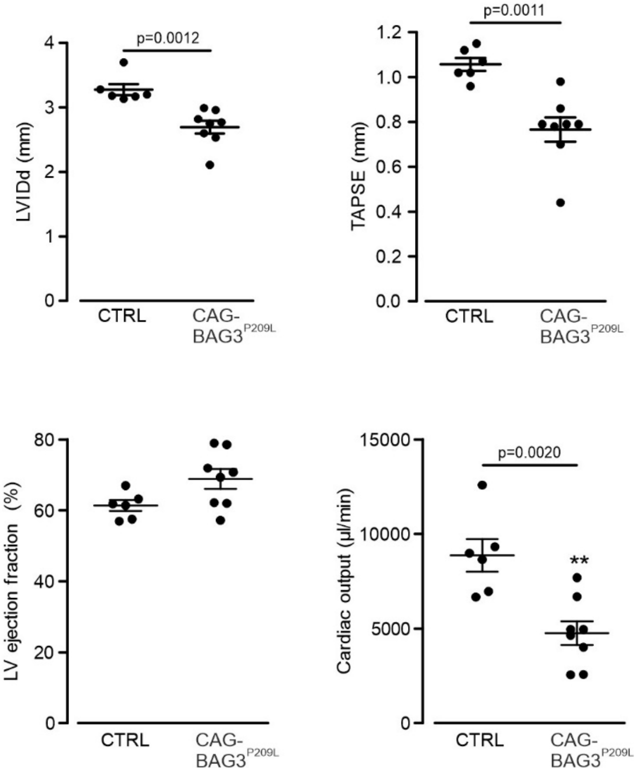

Supplementary Figure 6: Echocardiographic analysis of CAG-BAG3^{P209L} and CTRL-mouse hearts

a LVIDd (Left ventricular internal diameter end diastole), TAPSE (tricuspid annular plane systolic excursion), LV ejection fraction, and cardiac output were determined from parasternal M-Mode sections of the left ventricle. Mean ± SEM. n = 6 CTRL and 8 CAG-BAG3^{P209L} mice. Two-sided Student´s T-test. CTRL = control mice (siblings of CAG-BAG3^{P209L} mice, which are either WT, PGK-Cre, or CAG-flox-hBAG3^{P209L}).

a

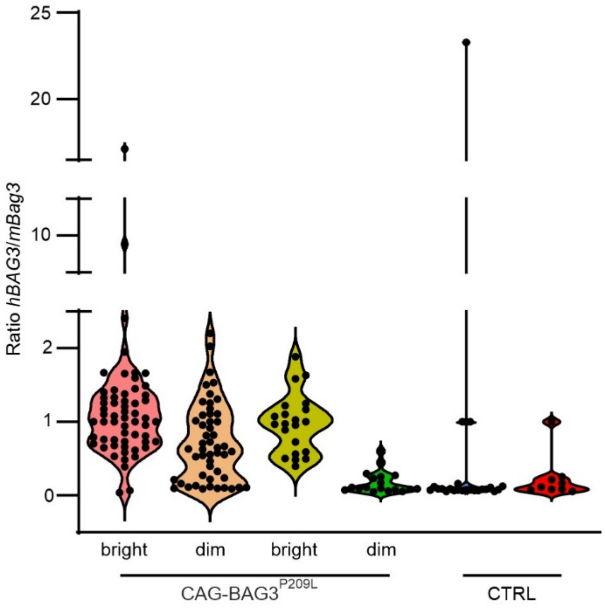

b

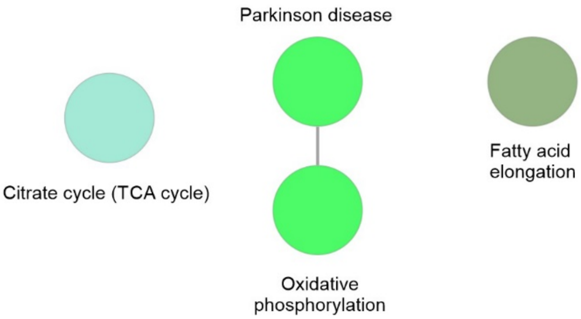

Supplementary Figure 7: scRNA-Seq analysis of CAG-BAG3^{P209L}- and CTRL-mouse hearts
a Ratio of hBAG3 to mBag3 mRNA in single CMs from 2-week-old CAG-BAG3^{P209L} and CTRL mice, as determined by single-cell RNA-Seq. CMs were subdivided into groups with bright and dim eGFP-expression, which was determined by a fluorescence macroscope. **b** KEGG terms of downregulated genes from 2-week-old homo CAG-BAG3^{P209L}compared to CTRL mice as determined by single-cell RNA-Seq. CTRL = control mice (siblings of CAG-BAG3^{P209L}-mice, which are either WT, PGK-Cre, or CAG-flox-hBAG3^{P209L}).

a

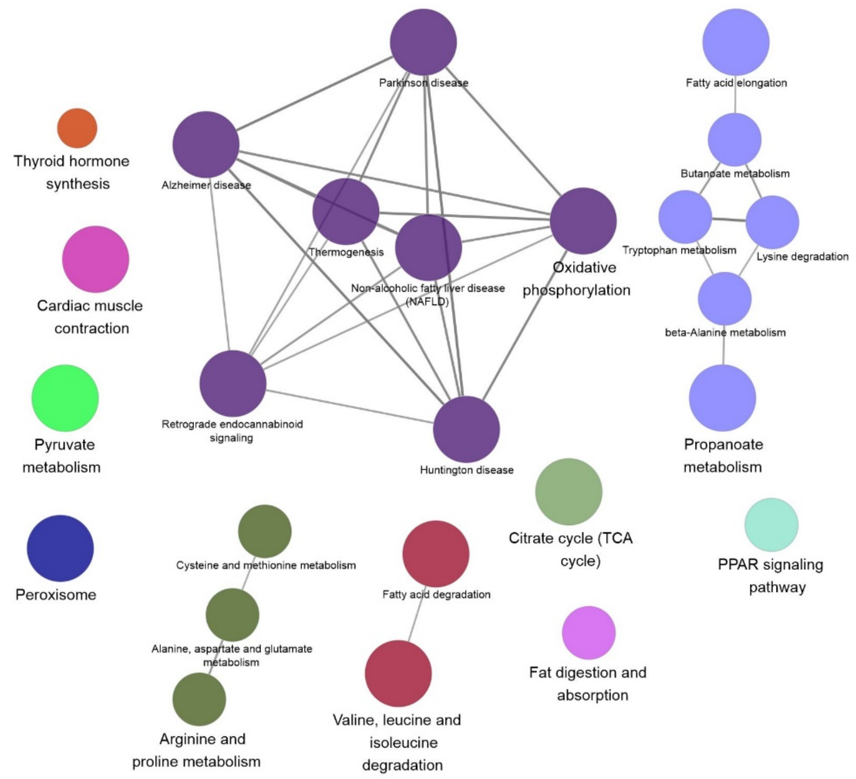

b

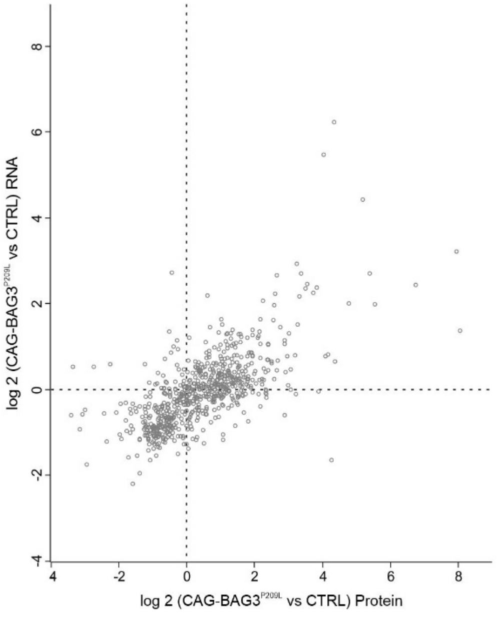

Supplementary Figure 8: Proteome analysis of 5-week-old CAG-BAG3^{P209L-} and CTRL-mouse hearts

a KEGG term analysis of proteins with significantly reduced abundance in 5-week-old CAG-BAG3^{P209L} mouse hearts compared to CTRL. **b** Correlation of protein and transcript abundance in

5-week-old CAG-BAG3^{P209L} mice hearts compared to CTRL. CTRL = control mice (siblings of CAG-BAG3^{P209L}-mice, which are either WT, PGK-Cre, or CAG-flox-hBAG3^{P209L}).

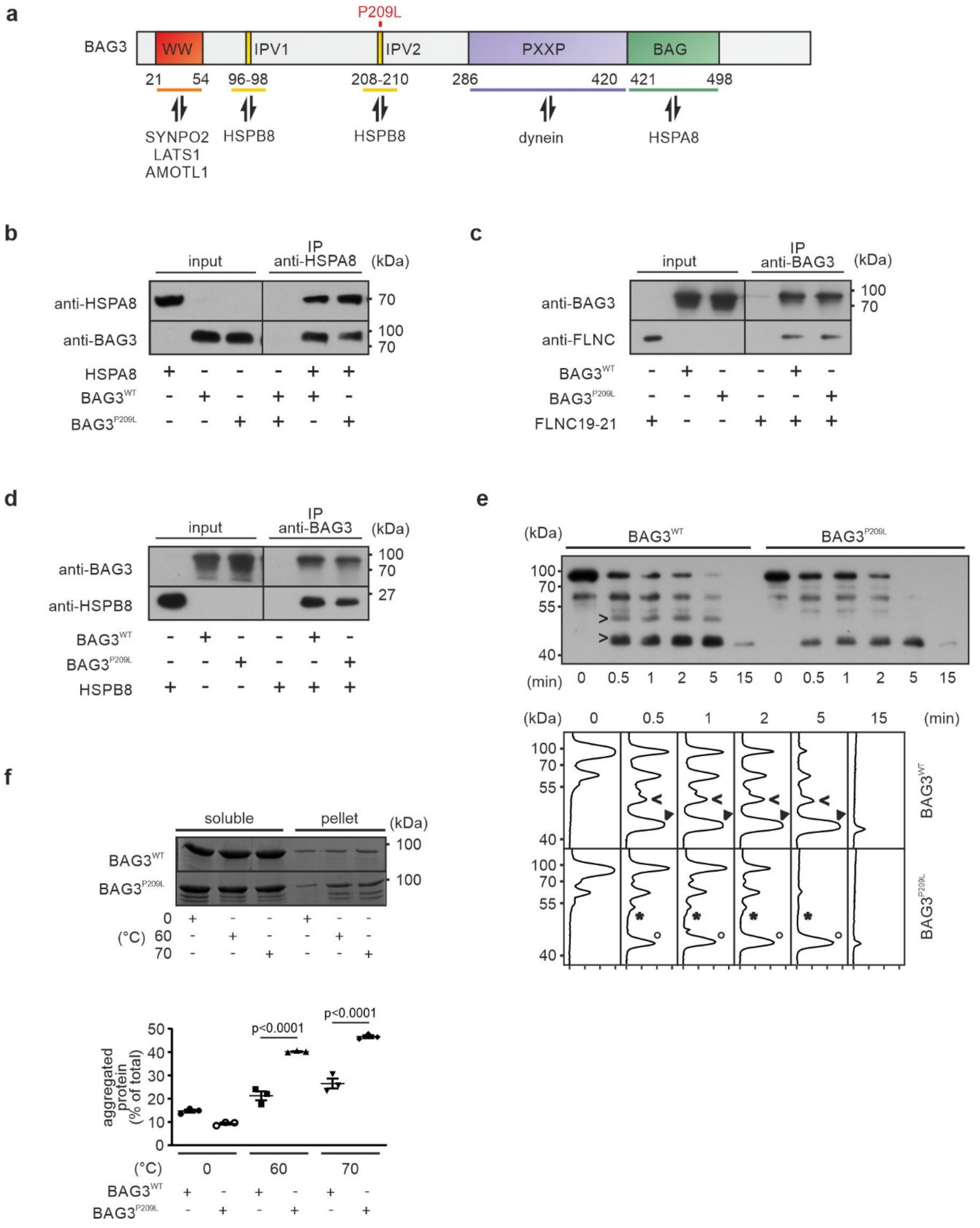

Supplementary Figure 9: Binding properties of hBAG3^{P209L} are unaltered *in vitro*, but hBAG3^{P209L} is more prone to denaturation compared with hBAG3^{WT}

a Schematic representation of the domain structure of human BAG3 and interactions with proteins involved in proteostasis. **b** Purified HSPA8 was immobilized on protein-G-sepharose with a specific antibody and incubated with purified hBAG3^{WT} or hBAG3^{P209L} as indicated. After extensive washing protein interactions were analyzed by western blotting. As negative control,

proteins were incubated with the affinity matrix alone. The left panel shows 5% of the input. **c** Purified $hBAG3^{WT}$ or $hBAG3^{P209L}$ was immobilized on protein-G-sepharose with a specific antibody and incubated with a purified FLNC-fragment containing the hBAG3 interaction site. After extensive washing protein interactions were analyzed by western blotting. As negative control, the FLNC fragment was incubated with the affinity matrix alone. The left panel shows 5% of the input. **d** Purified $hBAG3^{WT}$ or $hBAG3^{P209L}$ was immobilized on protein-G-sepharose with a specific antibody and incubated with purified HSPB8. After extensive washing protein interactions were analyzed by western blotting. As negative control, HSPB8 was incubated with the affinity matrix alone. The left panel shows 5% of the input. **b-d** The experiments were repeated three times from five independent biological replicates with similar results. **e** Purified bacterially expressed $hBAG3^{WT}$ or $hBAG3^{P209L}$ was incubated with trypsin at the indicated temperatures. Differences between partially digested proteins were visualized by western blotting with BAG3-antibody (open arrowheads). Partial digestion patterns were visualized and the apparent disparities hinting towards an altered conformation of $hBAG3^{P209L}$ marked (open arrowhead ≠ asterisk, solid arrowhead ≠ circle). **f** Purified $hBAG3^{WT}$ or $hBAG3^{P209L}$ was incubated at the indicated temperatures and denatured protein subsequently sedimented by centrifugation. Equivalent volumes of supernatant and pellet fractions were analyzed by immunoblotting and the results quantified. $hBAG3^{P209L}$ showed a significantly increased tendency to aggregate upon heat stress. Mean ± SEM. n= 3 experiments per group. One-way ANOVA.

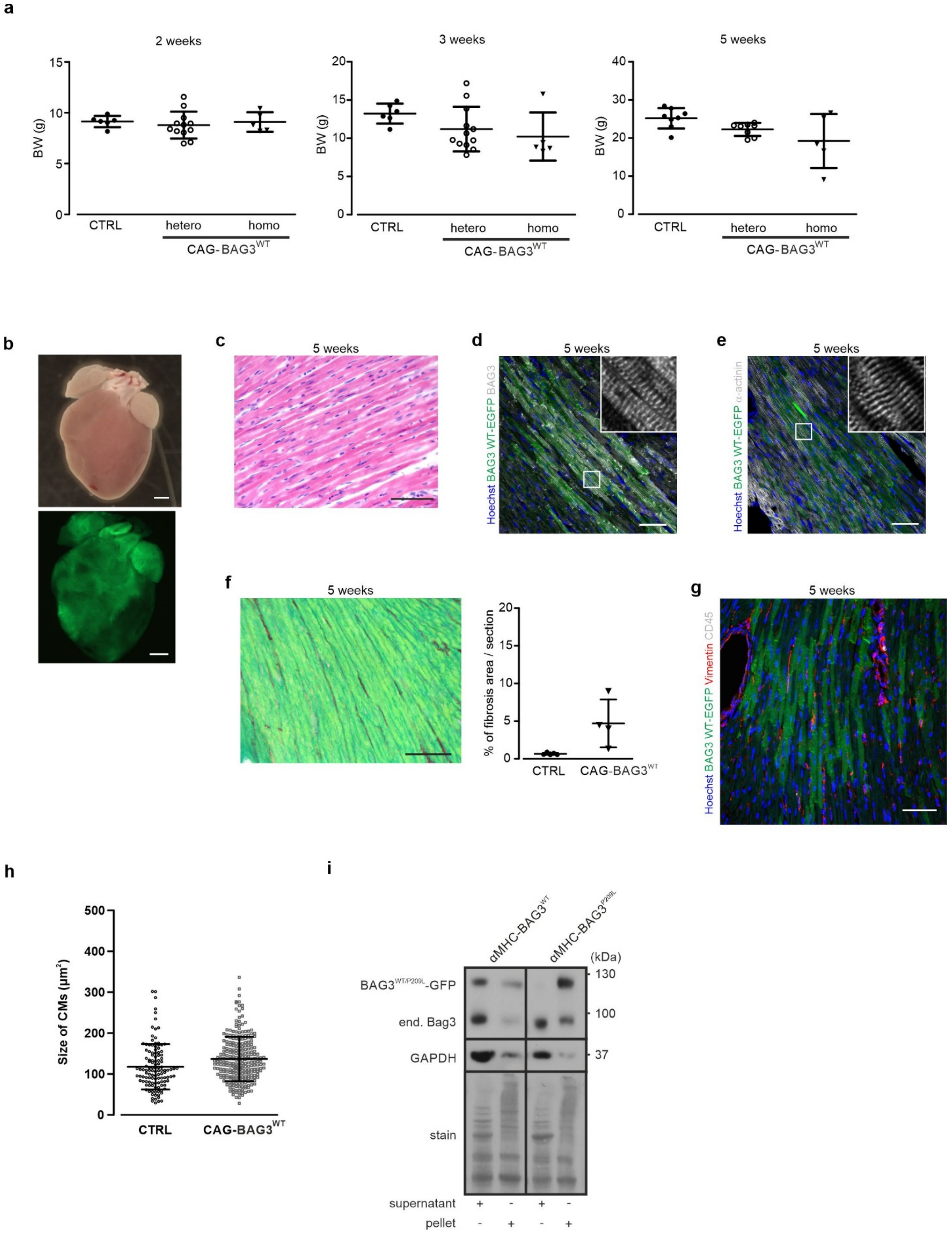

Supplementary Figure 10: Characterization of CAG-BAG3^{WT}-eGFP mice

a Body weight (BW) was unchanged in heterozygous and homozygous CAG-BAG3^{WT}-eGFP mice

in 2- (n = 6 CTRL, 12 heterozygous and 5 homozygous CAG-BAG3^{WT}-eGFP mice), 3- (n = 6 CTRL, 12 heterozygous and 5 homozygous CAG-BAG3^{WT}-eGFP mice), and 5-week-old (n = 8 CTRL, 8 heterozygous and 5 homozygous CAG-BAG3^{WT}-eGFP mice); Mean ± SEM. One-way ANOVA. **b** Representative heart from a 5-week-old homozygous CAG-BAG3^{WT} mouse displaying homogenous expression of the transgene. Scale bar: 1 mm. **c** HE-staining of hearts from 5-week-old homozygous CAG-BAG3^{WT} mice. Scale bar: 100 µm. **d**,**e** Section of a heart from a 5-week-old CAG-BAG3^{WT} mouse was stained for BAG3 **d** and α-actinin **e**. Scale bars: 50 µm. **b-e** The experiments were repeated three times from three independent biological replicates with similar results. **f** Analysis of cardiac fibrosis in a 5-week-old homozygous CAG-BAG3^{WT}-mouse by Sirius red and Fast green staining. Quantification of the fibrotic area in homozygous CAG-BAG3^{WT} (n = 4) and CTRL (n = 5) mice. Mean ± SEM. Two-sided Student´s T-test. Scale bar: 50 µm. **g** Section of heart from a 5-week-old CAG-BAG3^{WT} mouse was stained for vimentin (red) and CD45 (white). Scale bar: 50 µm. The experiments were repeated three times from three independent biological replicates with similar results. **h** Quantification of CM square area in cross-sections from hearts from CTRL and homozygous CAG-BAG3^{WT} mice at 5 weeks of age. Mean ± SEM. n = 128 CTRL and 259 CAG-BAG3^{WT} CMs. Two-sided Student´s T-test. CTRL = control mice (siblings of CAG-BAG3^{WT}-mice, which are either WT, PGK-Cre, or CAG-flox-hBAG3^{WT}). **i** Protein extracts from hearts of 10-week-old transgenic mice were prepared and analyzed by differential centrifugation and subsequent immunoblotting. hBAG3^{WT}-eGFP and murine BAG3 were mainly found in the supernatant (soluble fraction), while hBAG3^{P209L}-eGFP was predominantly found in the insoluble pellet fraction and seemed to have also sequestered wild-type endogenous BAG3 into the insoluble fraction. GAPDH served as control for successful separation of the fractions. The experiments were repeated two times from two independent biological replicates with similar results.

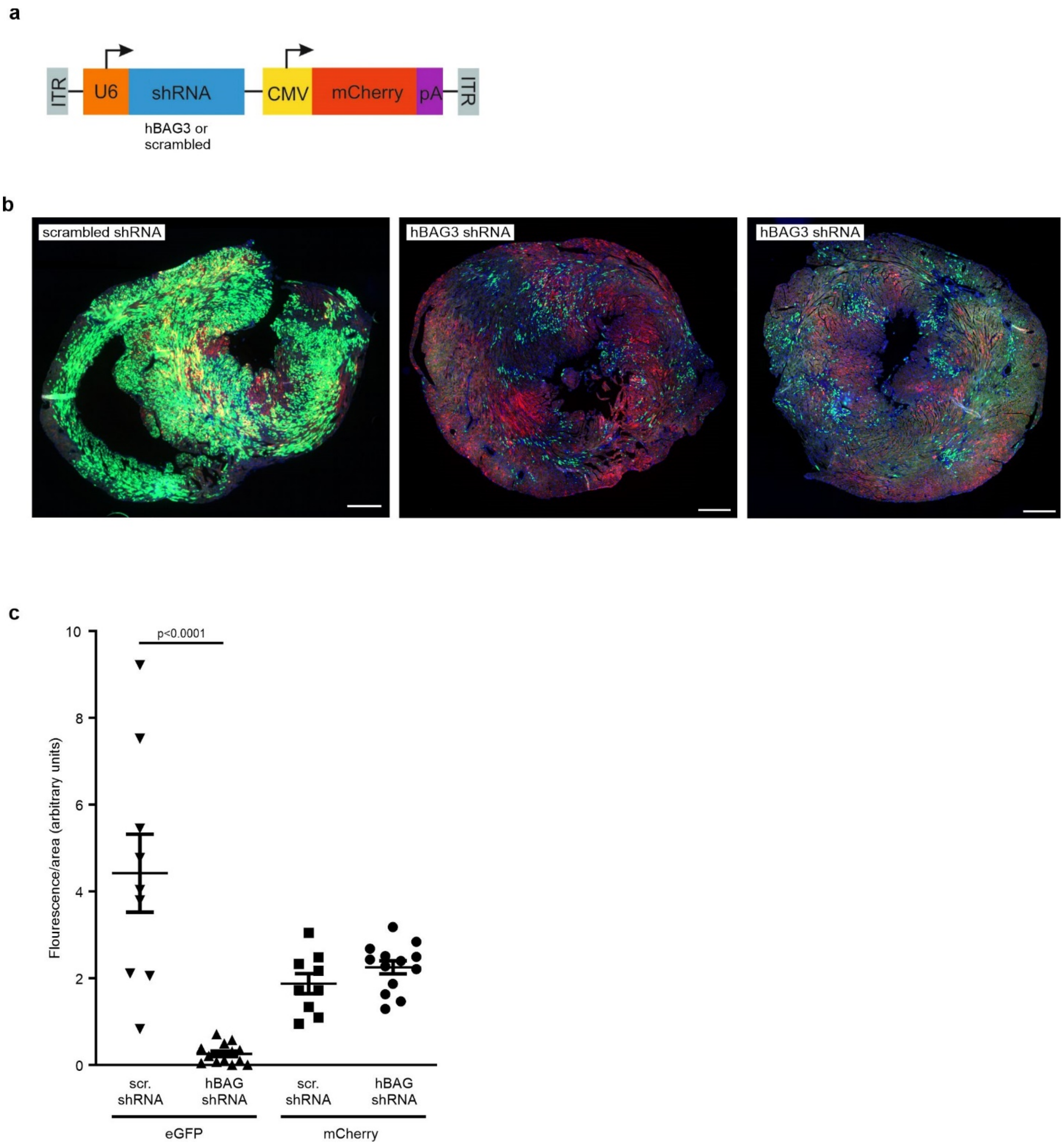

Supplementary Figure 11: AAV-based gene therapy approach of shRNA-mediated knockdown of hBAG3^{P209L}

a Scheme of recombinant AAV encoding either hBAG3- or scrambled shRNA under control of the U6 promoter; mCherry is under control of the CMV promoter and inserted between inverted terminal repeats (ITR). pA = polyA tail. **b** Cardiac cross-sections from AAV/rh10 treated CAG-BAG3^{P209L} mice at P37: hBAG3^{P209L}-eGFP expression and aggregates were reduced after treatment with AAV/rh10 hBAG3 shRNA. The reduction of hBAG3^{P209L}-eGFP expression in treated hearts showed some variability. Scale bars: 500 µm. **c** Quantification of eGFP and mCherry fluorescence intensities in cardiac sections after treatment with AAV/rh10 hBAG3 or scrambled shRNA. Mean

± SEM. n=9 scr. shRNA and 13 hBAG3 shRNA treated mice. Two-sided Student´s T-test. scr. shRNA = scrambled shRNA.

a

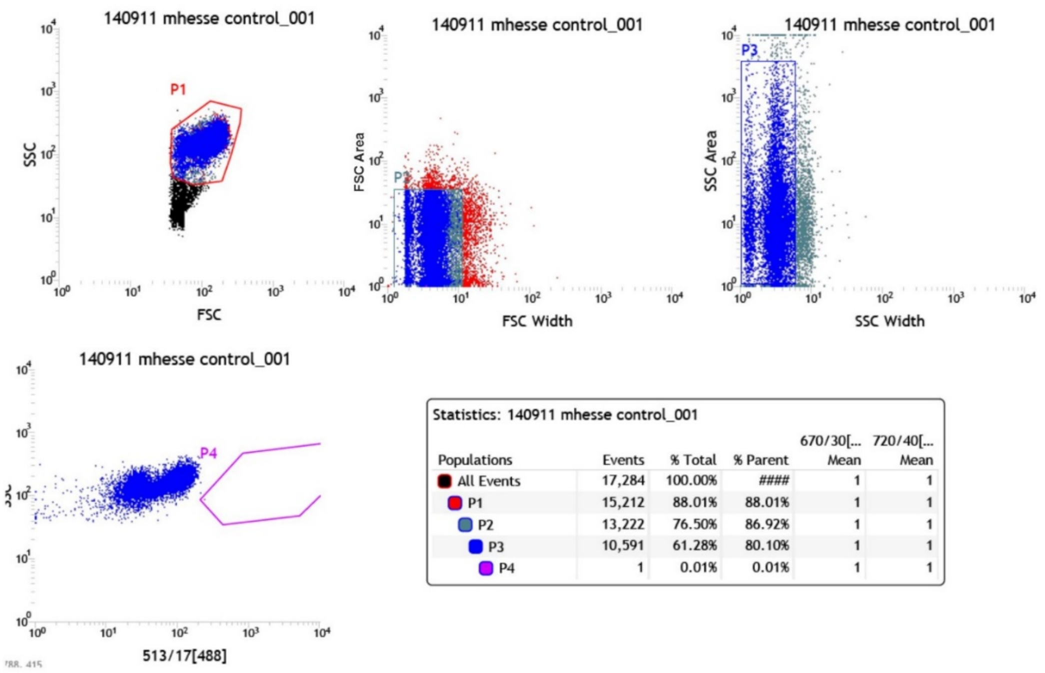

Statistics: 140911 mhesse control_001

Populations	Events	% Total	% Parent	670/30[... Mean	720/40[... Mean
All Events	17,284	100.00%	####	1	1
P1	15,212	88.01%	88.01%	1	1
P2	13,222	76.50%	86.92%	1	1
P3	10,591	61.28%	80.10%	1	1
P4	1	0.01%	0.01%	1	1

b

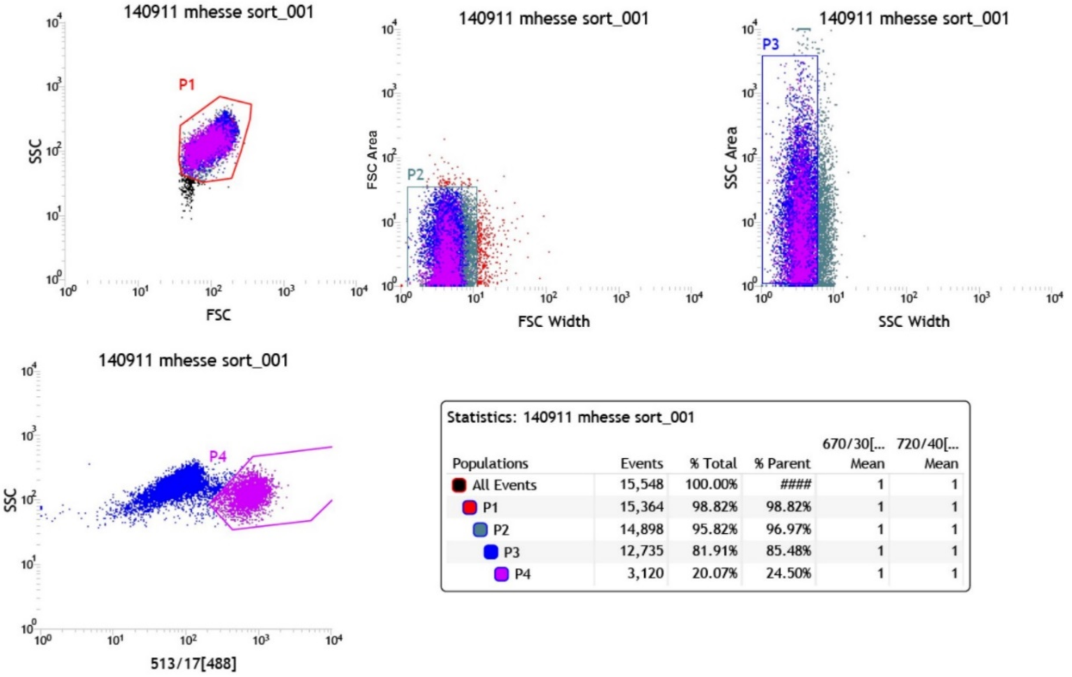

Statistics: 140911 mhesse sort_001

Populations	Events	% Total	% Parent	670/30[... Mean	720/40[... Mean
All Events	15,548	100.00%	####	1	1
P1	15,364	98.82%	98.82%	1	1
P2	14,898	95.82%	96.97%	1	1
P3	12,735	81.91%	85.48%	1	1
P4	3,120	20.07%	24.50%	1	1

Supplementary Figure 12: Gating strategy for sorting eGFP-positive CMs from αMHC-BAG3^{P209L} Langendorff-dissociated hearts

a Gating strategy established with cells from a Langendorff-dissociated heart of a wild-type mouse.

b Gating strategy of a sort for eGFP-positive CMs from a Langendorff-dissociated heart from a αMHC-BAG3^{P209L} mouse.

Supplementary Table 1: Echocardiographic parameters

Parameter	Control	BAG3 TG	P value
**Ejection fraction (%)**	61.3 ± 1.4	68.8 ± 2.6	0.0523
**LVPWd (mm)**	0.61 ± 0.03	0.66 ± 0.05	0.4531
**LVIDd (mm)**	3.28 ± 0.61	2.69 ± 0.09	0.0012
**Stroke volume (µl)**	21.7 ± 1.5	13.6 ± 1.2	0.0021
**Mitral valve E/A**	1.63 ± 0.11	2.79 ± 0.53	0.1114
**RVIDd (mm)**	1.28 ± 0.08	1.22 ± 0.08	0.6310
**PAT (ms)**	26.7 ± 1.9	18.9 ± 1.4	0.0096
**PET (ms)**	65.7 ± 1.5	71.0 ± 1.9	0.0747
**PAT / PET**	0.41 ± 0.03	0.27 ± 0.02	0.0035
**TAPSE (mm)**	1.06 ± 0.03	0.77 ± 0.05	0.0011
**Heart Rate (bpm)**	406.2 ± 16.4	343.6 ± 21.1	0.0475

LVIDd, left ventricular inner diameter during diastole; LVPWd, left ventricular posterior wall during diastole; PAT, pulmonary artery acceleration time; PET, pulmonary artery ejection time; RVIDd, right ventricular inner diameter during diastole; TAPSE, tricuspid annular plane systolic excursion; bpm, beats per minute. Mean ± SEM. n = 6 CTRL and 8 CAG-BAG3^{P209L} mice. Two-sided Student´s T-test.

Supplementary Table 2: Primers used for genotyping of transgenic mice

Name	Sequence	Purpose
BAG3*for*	5´-ATGAACTCCAGCCCAGCAAC-3´	Genotyping of αMHC-BAG3^{WT}-eGFP and αMHC-

		$BAG3^{P209L}$-eGFP transgenic mice
BAG3*rev*	5´-CCGCTTTACTTGTACAGCTC-3´	Genotyping of αMHC-$BAG3^{WT}$-eGFP and αMHC-$BAG3^{P209L}$-eGFP transgenic mice
CAG2-fw	5´-TTCGGCTTCTGGCGTGTGAC-3´	Genotyping of Tg(CAG-flox-$hBAG3^{WT}$-eGFP) and Tg(CAG-flox-$hBAG3^{P209L}$-eGFP) transgenic mice
BAG3-h-spez.tev	5´-GCACAGGAATGGGAATGTAG-3´	Genotyping of Tg(CAG-flox-$hBAG3^{WT}$-eGFP) and Tg(CAG-flox-$hBAG3^{P209L}$-eGFP) transgenic mice
Pgk1_for	5´-GCTGTTCTCCTCTTCCTCATC TCC-3´	Genotyping of PGK-Cre mice
Int-Cre_rev	5´-TCCATGAGTGAACGAACCTGG TCG-3´	Genotyping of PGK-Cre mice
