# Supplementary material for: Overexpression of human BAG3P209L in mice causes restrictive cardiomyopathy
Source: Nat Commun. 2021 Jun 11;12:3575. doi: 10.1038/s41467-021-23858-7 (PMC8196106; doi:10.1038/s41467-021-23858-7)
Supplement: Supplementary file 3 — Description of Additional Supplementary Files [file 41467_2021_23858_MOESM3_ESM.docx]

**Description of Additional Supplementary Files**

**File name: Supplementary Data 1.**

**Description:** Differentially expressed genes from single cell RNA-seq analysis by the FindMarkers function (two-sided non-parametric Wilcoxon rank sum test). p_val: p_val (unadjusted); avg_logFC : log fold-change of the average expression between the two groups. Positive values indicate that the gene is more highly expressed in the first group. pct.1: The percentage of cells where the gene is detected in the first group. pct.2: The percentage of cells where the gene is detected in the second group. p_val_adj: Adjusted p-value, based on Bonferroni correction using all genes in the dataset.

**File name: Supplementary Data 2.**

**Description:** ClueGO (two-sided hypergeometric test) was used to find over-represented GO terms in the ‘biological process’, and ‘KEGG pathways’ categories with a significance interval of <0.05. Bonferroni step down correction was performed for multiple testing-controlled P values.

**File name: Supplementary Data 3.**

**Description:** Differentially expressed genes from tissue RNA-seq analysis as determined by DeSeq2 (Two-sided Wald test statistic). Base mean: mean normalised counts, averaged over all samples from both conditions. log2(FC): the logarithm (to basis 2) of the fold change (See the note in inputs section). StdErr: standard error estimate for the log2 fold change estimate. Wald-Stats: Wald statistic. P-value: p value for the statistical significance of this change. P-adj: p value adjusted for multiple testing with the Benjamini-Hochberg procedure which controls false discovery rate (FDR).

**File name: Supplementary Data 4.**

**Description:** ClueGO (two-sided hypergeometric test) was used to find over-represented GO terms in the ‘biological process’, and ‘KEGG pathways’ categories with a significance interval of <0.05. Bonferroni step down correction was performed for multiple testing-controlled P values.

**File name: Supplementary Data 5.**

**Description:** Quantitative proteome analysis from 2-week-old mice. The "protein.txt" MaxQuant output file (available with the raw data as proteomeXchange dataset PXD021165) was loaded into Perseus, data restricted to proteins quantified in at least 4 animals of one genotype (4 valid LFQ values in at least one group) before imputation of missing values and two-sided Student’s t-test with Benjamini-Hochberg FDR <0.05. Columns L to W list LFQ values determined by MaxQuant before imputation and statistical analysis. (Note that the quantification of BAG3P209L_GFP in wt is an artefact from the employed standard settings which allow contribution of "shared" peptides to the majority protein, resulting in attribution of BAG3 wt peptides to BAG3P209L).

**File name: Supplementary Data 6.**

**Description:** Quantitative proteome analysis from 5-week-old mice. The "protein.txt" MaxQuant output file (available with the raw data as proteomeXchange dataset PXD021165) was loaded into Perseus, data restricted to proteins quantified in at least 4 animals of one genotype (4 valid LFQ values in at least one group) before imputation of missing values and two-sided Student’s t-test with Benjamini-Hochberg FDR <0.05. Columns L to W list LFQ values determined by MaxQuant before imputation and statistical analysis. (Note that the quantification of BAG3P209L in wt is an artefact from the employed standard settings which allow contribution of "shared" peptides to the majority protein, resulting in attribution of BAG3 wt peptides to BAG3P209L).

**File name: Supplementary Movie 1.**

**Description:** Representative B-MODE and M-MODE recordings from parasternal short-axis view in CAG-BAG3^P209L^ mice.

**File name: Supplementary Movie 2.**

**Description:** Representative B-MODE and M-MODE recordings from parasternal short-axis view in CAG-BAG3^WT^ mice.
