# Supplementary material for: Overexpression of human BAG3P209L in mice causes restrictive cardiomyopathy
Source: Nat Commun. 2021 Jun 11;12:3575. doi: 10.1038/s41467-021-23858-7 (PMC8196106; doi:10.1038/s41467-021-23858-7)
Supplement: Supplementary file 12 — Reporting Summary [file 41467_2021_23858_MOESM12_ESM.pdf]

nature research

Corresponding author(s): Michael Hesse, Bernd K. Fleischmann

Last updated by author(s): Feb 18, 2021

Reporting Summary

Nature Research wishes to improve the reproducibility of the work that we publish. This form provides structure for consistency and transparency in reporting. For further information on Nature Research policies, see our Editorial Policies and the Editorial Policy Checklist.

Statistics

For all statistical analyses, confirm that the following items are present in the figure legend, table legend, main text, or Methods section.

n/a	Confirmed	
☐	☒	The exact sample size (*n*) for each experimental group/condition, given as a discrete number and unit of measurement
☐	☒	A statement on whether measurements were taken from distinct samples or whether the same sample was measured repeatedly
☐	☒	The statistical test(s) used AND whether they are one- or two-sided<br>*Only common tests should be described solely by name; describe more complex techniques in the Methods section.*
☒	☐	A description of all covariates tested
☐	☒	A description of any assumptions or corrections, such as tests of normality and adjustment for multiple comparisons
☐	☒	A full description of the statistical parameters including central tendency (e.g. means) or other basic estimates (e.g. regression coefficient) AND variation (e.g. standard deviation) or associated estimates of uncertainty (e.g. confidence intervals)
☐	☒	For null hypothesis testing, the test statistic (e.g. *F*, *t*, *r*) with confidence intervals, effect sizes, degrees of freedom and *P* value noted<br>*Give P values as exact values whenever suitable.*
☒	☐	For Bayesian analysis, information on the choice of priors and Markov chain Monte Carlo settings
☒	☐	For hierarchical and complex designs, identification of the appropriate level for tests and full reporting of outcomes
☒	☐	Estimates of effect sizes (e.g. Cohen's *d*, Pearson's *r*), indicating how they were calculated

Our web collection on statistics for biologists contains articles on many of the points above.

Software and code

Policy information about availability of computer code

Data collection	HyStar Software v3.2 for proteomics data acquisition<br>Odyssey Infrared Imaging Software v. 3.0 for acquisition of immunoblots<br>Image Lab v5.2.1 for immunoblot acquisition<br>Axiovision Rel. 4.8 Software for microscopical picture acquisition<br>BD FACS™ Sortware version 1.0.1.654 for acquisition of flow cytometry data<br>Vevo LAB software for acquisition of echocardiography data
Data analysis	GraphPad Prism V9.0.1 for statistical analysis<br>PS Power and Sample Size Calculations programm V3.1.6 for calculation of sample size for animal experiments<br>Odyssey Infrared Imaging Software v. 3.0 for quantification of immunoblots<br>Image Lab 5.2.1 for quantification of immunoblots<br>Perseus version 1.6.10.0 for analysis of label-free quantification (LFQ) data<br>MaxQuant version 1.6.6.0 and 1.6.10.43 for analysis of proteomics data<br>Fiji (ImageJ) version 1.47n software package for quantification of protein gels<br>Seurat V3.1 for analysis of single cell RNA-seq data<br>Galaxy platform version 2018 for analysis of tissue RNA-seq data<br>ClueGO v2.5.6 app in cytoscape v3.7.1 for gene ontology analysis<br>Axiovision Rel. 4.8 Software for picture analysis<br>BD FACS™ Sortware version 1.0.1.654 for analysis of flow cytometry data<br>Vevo LAB version 3.2.5 software for analysis of echocardiography data<br>DESeq2 version 2.11.40.6 for analysis of tissue RNA-seq data<br>RNA STAR version 2.7.2b for analysis of tissue RNA-seq data

featureCounts version 1.6.4+galaxy2 for analysis of tissue RNA-seq data
heatmap2 version 3.0.1 for analysis of tissue RNA-seq data
Volcano plot version 0.0.3 for analysis of tissue RNA-seq data

For manuscripts utilizing custom algorithms or software that are central to the research but not yet described in published literature, software must be made available to editors and reviewers. We strongly encourage code deposition in a community repository (e.g. GitHub). See the Nature Research guidelines for submitting code & software for further information.

Data

Policy information about availability of data

All manuscripts must include a data availability statement. This statement should provide the following information, where applicable:

- Accession codes, unique identifiers, or web links for publicly available datasets
- A list of figures that have associated raw data
- A description of any restrictions on data availability

MS data have been deposited to the ProteomeXchange Consortium via the PRIDE partner repository (https://www.ebi.ac.uk/pride/archive/projects/PXD021165) with the project accession number PXD021165. Single cell RNA-seq data have been deposited to the GEO database with the GEO accession number GSE166862 (https://www.ncbi.nlm.nih.gov/geo/query/acc.cgi?acc=GSE166862). Tissue RNA-seq data have been deposited to the SRA database with the BioProject accession number PRJNA700583 (https://www.ncbi.nlm.nih.gov/sra/PRJNA700583). Raw data have been provided as a source data file. Raw data have been provided as a sSource data for figure(s) [number(s)] are provided with the paperfile for figures 5f, 6a, 7b, and Supplementary figures 1c, 9b-f, and 10i. All other source data are provided upon request.

Field-specific reporting

Please select the one below that is the best fit for your research. If you are not sure, read the appropriate sections before making your selection.

[x] Life sciences   [ ] Behavioural & social sciences   [ ] Ecological, evolutionary & environmental sciences

For a reference copy of the document with all sections, see nature.com/documents/nr-reporting-summary-flat.pdf

Life sciences study design

All studies must disclose on these points even when the disclosure is negative.

Sample size	The minimal sample size was determined by a power analysis using PS Power and Sample Size Calculations programm V3.1.6 from Vanderbilt university (http://biostat.mc.vanderbilt.edu/PowerSampleSize) with the following assumptions: We are planning a study of a continuous response variable from independent control and experimental subjects with 1 control per experimental subject. In a previous study the response within each subject group was normally distributed with standard deviation 0.1. If the true difference in the experimental and control means is 0.2, we will need to study 5 experimental subjects and 5 control subjects to be able to reject the null hypothesis that the population means of the experimental and control groups are equal with probability (power) 0.8. The Type I error probability associated with this test of this null hypothesis is 0.05.
Data exclusions	We did not exclude data to reflect the biological variability in our transgenic mice, which is also seen in patients suffering from BAG3P209L myofibrillar myopathy.
Replication	The reproducibility of the experimental findings was verified by using at least n=3 mice for all experiments. All attemps at replication were successful. For experiments not involving mice at least 3 biological replicates and 2 technical replicates were used with similar results.
Randomization	Transgenic mice and controls were randomly assigned to their respective groups. Mice were age matched but not sex matched or littermate matched. For experiments not involving mice biological replicates were randomly assigned to their respective groups.
Blinding	The investigators were blinded to the gene therapy approach during the application of AAV. The researchers were not blinded to group allocation because the difference in size between transgenic mice and controls was too obvious.

Reporting for specific materials, systems and methods

We require information from authors about some types of materials, experimental systems and methods used in many studies. Here, indicate whether each material, system or method listed is relevant to your study. If you are not sure if a list item applies to your research, read the appropriate section before selecting a response.

Materials & experimental systems

n/a	Involved in the study
	☒ Antibodies
	☒ Eukaryotic cell lines
☒	Palaeontology and archaeology
	☒ Animals and other organisms
☒	Human research participants
☒	Clinical data
☒	Dual use research of concern

Methods

n/a	Involved in the study
☒	ChIP-seq
	☒ Flow cytometry
☒	MRI-based neuroimaging

Antibodies

Antibodies used

Anti-α-Actinin (Sigma Aldrich A7811, lot# 128M4812V), used at 1:400 IF
Anti-alpha B Crystallin (Enzo Life Science ADI-SPA-223D), used at 1:5000 WB
Anti-Bag3 (Proteintech 10599-1-AP, lot# 00054511), used at 1:5000 WB; 1:250 IF
Anti-CD45 (Millipore CBL3126, lot# LV1587428), used at 1:400 IF
Anti-Desmin (Dako M0760, lot# 20079872), used at 1:100 IF
Anti-Filamin C (Biogene), used at 1:1000 IF
Anti-HSPB6 (Abcam Ab13491), used at 1:2000 WB
Anti-HSPB7 (Abcam Ab150390, lot# GR105863-7), used at 1:1000 WB
Anti-HSPB8 (Abcam Ab151552, lot# GR112670-9), used at 1:1000 WB; 1:67 IF
Anti-HSPB8 (Cell Signaling Technology #3059), used at 1:1000 WB
Anti-LC3B (Thermo Fisher Scientific 70012, lot# GR112670-9), used at 1:1000 WB
Anti-mCherry (Novus Biologicals NBP2-25157, lot# 205091119), used at 1:5000 WB
Anti-p62 (Progen GP62-C, lot# 709151), used at 1:1000 WB
Anti-Titin (Max-Planck-Institute for Biophysical Chemistry, Göttingen, Germany, custom made), used at 1:50 IF
Anti-Titin (Eurogentec, custom made, TTN-PEVK), used at 1:100 EM
Anti-Vimentin (Millipore AB5733), used at 1:1000 IF
Anti-Endomucin (Santa Cruz sc65495, clone V.7c7), used at 1:200 IF
Anti-CD68 (eBioscience 14-0681-82, clone FA-11), used at 1:100 IF
Anti-cCasp3 (Cell Signaling Technology 9661L), used at 1:50 IF
Anti-HSPA8 (Enzo Life Sciences ADI-SPA-815-D, clone 1B5), used at 1:100 IF
Anti-HSPA8 (raised against purified rat HSPA8 following recombinant expression in insect cells, rabbit immunization and antibody purification were performed at BioGenes GmbH), used at 1:1000 WB
Anti-Synpo2 (Sigma Aldrich M9818), used at 1:100 IF
Anti-GAPDH (Calbiochem CB1001, clone 6C5, lot# 3167011), used at 1:1000 WB
Anti-pan MyHC (Developmental Studies Hybridoma Bank clone MF20), used at 1:100 WB
Anti-His (Serotec MCA1396), used at 1:1000 WB
Anti-MBP (Abcam ab23903), used at 1:1000 WB
Anti-beta-actin (Invitrogen MA1-140, lot #SE253380), used at 1:2000 WB
IRDye-800-conjugated goat anti-mouse secondary antibody (LI-COR Biosciences, 926-32210, Lot# C30509-01, Bad Homburg, Germany), used at 1:10000 WB
IRDye-800-conjugated goat anti-rabbit secondary antibody (LI-COR Biosciences, 926-32211, Lot# C30521-01, Bad Homburg, Germany, used at 1:10000 WB
Alexa 488 donkey anti-rabbit (Jackson ImmunoResearch 711-545-152, lot# 151331), used at 1:400 IF
Cy2 donkey anti-mouse (Jackson ImmunoResearch 715-225-151, lot# 108625), used at 1:400 IF
Cy2 donkey anti-rat (Jackson ImmunoResearch 712-225-153), used at 1:400 IF
Alexa 647 donkey anti-rat (Jackson ImmunoResearch 712-605-153, lot# 120620), used at 1:400 IF
Alexa 647 donkey anti-mouse (Jackson ImmunoResearch 715-605-101, lot# 152527), used at 1:400 IF
Cy3 donkey anti-rat (Jackson ImmunoResearch 712-165-153 lot# 143459), used at 1:400 IF
Cy3 donkey anti-mouse (Jackson ImmunoResearch 115-165-207, lot# 144073), used at 1:400 IF
Peroxidase-coupled goat anti-rabbit (Jackson ImmunoResearch 111-035-144, lot# 124305), used at 1:10000 WB
Cy3 donkey anti-rabbit (Jackson ImmunoResearch 711-165-152, lot# 0152679), used at 1:3000 WB
Alexa 647 donkey anti-rabbit (Jackson ImmunoResearch 711-605-152 und lot# 150965), used at 1:3000 WB

Validation

Anti-α-Actinin (Sigma Aldrich A7811) has been validated for use in immunohistochemistry and western blotting in mouse tissue, as indicated on the manufacturer's product page.
Anti-alpha B Crystallin (Enzo Life Science ADI-SPA-223D) has been validated for use in immunohistochemistry and western blotting in mouse tissue, as indicated on the manufacturer's product page.
Anti-Bag3 (Proteintech 10599-1-AP) has been validated for use in immunohistochemistry and western blotting in mouse tissue, as indicated on the manufacturer's product page.
Anti-CD45 (Millipore CBL1326) has been validated for use in immunohistochemistry and western blotting in mouse tissue, as indicated on the manufacturer's product page.
Anti-Desmin (Dako M0760) has been validated for use in immunohistochemistry and western blotting in mouse tissue, as indicated on the manufacturer's product page.

Anti-Filamin C (custom made) has been validated in the following publication: PMID: 20060297.
Anti-HSPB6 (Abcam Ab13491), has been validated for use in western blotting in rat and human tissue , as indicated on the manufacturer's product page. Validation in mice tissue in PMID: 28737513.
Anti-HSPB7 (Abcam Ab150390) has been validated for use in western blotting in mouse tissue, as indicated on the manufacturer's product page.
Anti-HSPB8 (Abcam Ab151552) has been validated for use in immunohistochemistry in mouse tissue, as indicated on the manufacturer's product page.
Anti-HSPB8 (Cell Signaling Technology #3059) has been validated for use in western blotting in mouse tissue, as indicated on the manufacturer's product page.
Anti-LC3B (Thermo Fisher Scientific 70012) has been validated for use in immunohistochemistry and western blotting in mouse tissue, as indicated on the manufacturer's product page.
Anti-mCherry (Novus Biologicals NBP2-25157) has been validated for use in western blotting in mouse tissue, as indicated on the manufacturer's product page.
Anti-p62 (Progen GP62-C) has been validated for use in immunohistochemistry and western blotting in mouse tissue, as indicated on the manufacturer's product page.
Anti-Titin (Max-Planck-Institute for Biophysical Chemistry, Göttingen, Germany, custom made) has been validated in the following publication: PMID: 2453516.
Anti-Titin (Eurogentec, custom made, TTN-PEVK) has been validated in the following publication: PMID: 23047121
Anti-Vimentin (Millipore AB5733) has been validated for use in immunohistochemistry in mouse tissue, as indicated on the manufacturer's product page.
Anti-Endomucin (Santa Cruz sc65495) has been validated for use in immunohistochemistry in mouse tissue, as indicated on the manufacturer's product page.
Anti-CD68 (eBioscience 14-0681-82) has been validated for use in immunohistochemistry in mouse tissue, as indicated on the manufacturer's product page.
Anti-cCasp3 (Cell Signaling Technology 9661L), has been validated for use in immunohistochemistry in mouse tissue, as indicated on the manufacturer's product page.
Anti-HSPA8 (Enzo Life Sciences ADI-SPA-815-D) has been validated for use in immunohistochemistry in mouse tissue, as indicated on the manufacturer's product page.
Anti-Synpo2 (Sigma Aldrich M9818) has been validated for use in immunohistochemistry in rat tissue, as indicated on the manufacturer's product page.
Anti-GAPDH (Calbiochem CB1001, clone 6C5, lot# 3167011) has been validated for use in western blotting in mouse tissue, as indicated on the manufacturer's product page.
Anti-pan MyHC (Developmental Studies Hybridoma Bank clone MF20) has been validated for use in western blotting in mouse tissue, as indicated on the manufacturer's product page.
Anti-His (Serotec MCA1396) has been validated for use in western blotting to detect and purify histidine-tagged proteins expressed in mammalian and non-mammalian cell lines, as indicated on the manufacturer's product page.
Anti-MBP (Abcam ab23903) has been validated for use in western blotting to recognize proteins expressed with an MBP tag, as indicated on the manufacturer's product page.
Anti-HSPA8 (custom made) has been validated in the following publication: PMID: 23434281.
Anti-beta-actin (Invitrogen MA1-140) has been validated for use in western blotting in mouse tissue, as indicated on the manufacturer's product page.
IRDye-800-conjugated goat anti-mouse secondary antibody (LI-COR Biosciences, 926-32210) has been validated for use in western blotting, as indicated on the manufacturer's product page.
IRDye-800-conjugated goat anti-rabbit secondary antibody (LI-COR Biosciences, 926-32211) has been validated for use in western blotting, as indicated on the manufacturer's product page.

Eukaryotic cell lines

Policy information about cell lines

Cell line source(s)	The G4-ES cell line was obtained from the lab of Andras Nagy, Toronto, Canada which is the original source for this cell line (see Acknowledgement).
Authentication	The G4-ES cell line was authenticated by using it to generate all transgenic mouse lines in this study.
Mycoplasma contamination	All cell lines tested negative for mycoplasma contamination.
Commonly misidentified lines (See ICLAC register)	No commonly misidentified cell lines were used in this study.

Animals and other organisms

Policy information about studies involving animals; ARRIVE guidelines recommended for reporting animal research

Laboratory animals	We used male and female 2-5 weeks old transgenic PGK-Cre/CAG-flox-hBAG3WT-eGFP, and PGK-Cre/CAG-flox-hBAG3P209L-eGFP and littermate control mice (Mus musculus) and male and female 10 weeks old transgenic αMHC-BAG3WT-eGFP, αMHC-BAG3P209L-eGFP and littermate control mice (Mus musculus) on a mixed 129S6/SvEvTac x C57BL/6Ncr x CD-1 genetic background.
Wild animals	The study did not involve wild animals.
Field-collected samples	The study did not involve samples collected from the field.

Ethics oversight	Animal experiments were approved by the responsible governmental animal care and use office, the Landesamt für Natur, Umwelt und Verbraucherschutz, LANUV (84-02.04.2012A146 and 81-02.04.2019.A062)

Note that full information on the approval of the study protocol must also be provided in the manuscript.

Flow Cytometry

Plots

Confirm that:

- [x] The axis labels state the marker and fluorochrome used (e.g. CD4-FITC).
- [x] The axis scales are clearly visible. Include numbers along axes only for bottom left plot of group (a 'group' is an analysis of identical markers).
- [x] All plots are contour plots with outliers or pseudocolor plots.
- [x] A numerical value for number of cells or percentage (with statistics) is provided.

Methodology

Sample preparation	Hearts from transgenic aMHC-BAG3P209L mice and control mice were dissociated by Langendorff perfusion.
Instrument	inFlux v7 Sorter cytometer (Becton-Dickinson).
Software	BD FACS™ Sortware version 1.0.1.654 software was used for analysis.
Cell population abundance	The abundance of EGFP-positive cardiomyocytes after sorting was not determined but was assumed to be >90% based on the gating strategy.
Gating strategy	Gating for eGFP was set using CMs from CAG-eGFP transgenic mouse hearts autofluorescence was detected by using CMs from wildtype mice.

- [x] Tick this box to confirm that a figure exemplifying the gating strategy is provided in the Supplementary Information.
